# Role of mtDNA Haplogroups in the Prevalence of Osteoarthritis in Different Geographic Populations: A Meta-Analysis

**DOI:** 10.1371/journal.pone.0108896

**Published:** 2014-10-23

**Authors:** Jin-Ming Shen, Lei Feng, Chun Feng

**Affiliations:** 1 Department of Orthopedics, the First Affiliated Hospital of Zhejiang Chinese Medicine University, Hangzhou, Zhejiang, China; 2 Department of Reproductive Endocrinology, Women's Hospital, School of Medicine, Zhejiang University, Hangzhou, China; IIBB/CSIC/IDIBAPS, Spain

## Abstract

**Background:**

Osteoarthritis (OA) is the most common form of arthritis and has become an increasingly important public-health problem. However, the pathogenesis of OA is still unclear. In recent years, its correlation with mtDNA haplogroups attracts much attention. We aimed to perform a meta-analysis to investigate the association between mtDNA haplogroups and OA.

**Methods:**

Published English or Chinese literature from PubMed, Web of Science, SDOS, and CNKI was retrieved up until April 15, 2014. Case-control or cohort studies that detected the frequency of mtDNA haplogroups in OA patients and controls were included. The quality of the included studies was evaluated by the Newcastle-Ottawa Scale (NOS) assessment. A meta-analysis was conducted to calculate pooled odds ratio (OR) with 95% confidence interval (CI) through the random or fixed effect model, which was selected based on the between-study heterogeneity assessed by Q test and *I^2^* test. Subgroup analysis was performed to explore the origin of heterogeneity.

**Results:**

A total of 6 case-control studies (10590 cases and 7161 controls) with an average NOS score of 6.9 were involved. For the analysis between mtDNA haplogroup J and OA, random model was selected due to high heterogeneity. No significant association was found initially (OR = 0.73, 95%CI: 0.52–1.03), however, once any study from UK population was removed the association emerged. Further subgroup analysis demonstrated that there was a significant association in Spain population (OR = 0.57, 95%CI: 0.46–0.71), but not in UK population. Also, subgroup analysis revealed that there was a significant correlation between cluster TJ and OA in Spain population (OR = 0.70, 95%CI: 0.58–0.84), although not in UK population. No significant correlation was found between haplogroup T/cluster HV/cluster KU and OA.

**Conclusions:**

Our current meta-analysis suggests that mtDNA haplogroup J and cluster TJ correlate with the risk of OA in Spanish population, but the associations in other populations require further investigation.

## Introduction

Osteoarthritis (OA), also known as degenerative arthritis and proliferative arthritis, is a chronic progressive disease characterized with degenerative changes and secondary hyperosteogeny of articular cartilage, which leads to pain and dysfunction of joints. OA is the most common form of arthritis, rare before 40 years old, and rising steeply with age. In fact, OA occurs in more than 50% of the population older than 65 years old [1], and is the leading cause of global disability [2]. Due to the aged tendency of population, it is estimated that the population suffering with OA may double in the next three decades [3]. So far, the pathogenesis of OA is still unclear, but it is now accepted that genetic factors play a role in it. Familial aggregation [4] and classical twin [5] studies have both demonstrated the key contribution of genetic susceptibility in OA.

The mitochondrion is a membrane-enclosed organelle that converts dietary intake into ATP by oxidative phosphorylation (OXPHOS) and meanwhile produces reactive oxygen species (ROS) which may lead to oxidative damage of the cell [6]. A great amount of evidence has suggested that mitochondrial degeneration may be associated with some human diseases, including Alzheimer disease, Parkinson disease, Huntington disease, cancer and some aging processes [7]–[9]. Moreover, mitochondrial dysfunction may contribute to the damage in articular cartilage and participate in the occurrence and progression of OA [10].

Mitochondrial DNA (mtDNA) encodes 13 of their mitochondrial proteins [11]. According to the various single nucleotide polymorphisms (SNPs) in mtDNA coding regions, related groups of mtDNA haplogroups are defined [12]. Recently, the association between mtDNA haplogroups and OA has become a hot area of research. Some studies reported that specific mtDNA haplogroups were correlated with the development and progression of OA [13]–[17], while others detected no significant association [18]. The discrepancy may be due to many factors, such as different ethnicity, geological variation and relatively small sample size.

Provided with the studies published in the past year, it is possible to collect sufficient data to explore the authentic interrelation between mtDNA haplogroups and OA. Therefore, in an attempt to ascertain the correlation of mtDNA haplogroups and risk of OA, we performed a meta-analysis to strengthen the statistical power of individual studies.

## Materials and Methods

This meta-analysis was developed according to the PRISMA guidelines (Checklist S1). A protocol was registered in PROSPERO, with registration number: CRD42014009640.

### Search strategy

Relevant studies published in English or Chinese were identified by a computerized search via 4 databases, including PubMed, Web of Science, Elsevier ScienceDirect (SDOS), and China National Knowledge Infrastructure (CNKI). The search strategy involved the use of the following key words: *Osteoarthritis* AND (*mtDNA* OR “*mitochondrial DNA*”) AND (*haplogroup* OR *haplotype* OR *genotype* OR “*genetic predisposition*” OR *SNP* OR polymorphism* OR variant* OR “*genetic susceptibility*” OR *genetics* OR *allele*), with details in Methods S1. The reference lists of retrieved articles were hand-searched to collect other relevant studies. The non-published studies were searched in the database of OpenSIGLE (System for Information on Grey literature in Europe, http://www.opengrey.eu). The literature in these databases from inception to April 15, 2014 was searched by two separate reviewers. If more than one article was published with the same case series, only the study with the largest sample size was selected.

### Inclusion and exclusion criteria

The studies to meet the following inclusion criteria were screened: (1) evaluating the association between haplogroups of mtDNA and the risk of OA; (2) case-control or cohort design; (3) sufficient data provided to calculate odds ratio (OR) with the corresponding 95% confidence interval (95% CI). Studies were excluded if they: (1) explored the correlation between haplogroups of mtDNA and the progression of OA, not the prevalence; (2) focused on other mtDNA mutation except haplogroups. All identified studies were reviewed independently by two investigators to determine if an individual study should be included in this study and the discrepancies were resolved by discussion.

### Data extraction

The following data were extracted from each study by two investigators independently: (1) name of the first author; (2) year of publication; (3) country; (4) ethnicity; (5) gender of subjects; (6) age of subjects; (7) diagnosis criteria; (8) types of OA; (9) methods of genotype; (10) conclusion of the study; (11) study type; (12) controlled confounders; and (13) numbers of genotypes in cases and controls. We contacted investigators for additional information when necessary.

### Quality assessment of study

The quality of all the included studies was evaluated independently by two authors using the Newcastle-Ottawa Scale (NOS) assessment [19]. Any disagreements were addressed by discussion. The NOS assessment consists of three parameters of quality appraisal: selection of cases and controls, comparability of case and control groups, and ascertainment of exposure.

### Statistical analysis

The degree of between-study heterogeneity was determined by Q test and *I^2^* test. A *P* value (Q test) ≥0.05 or *I^2^*<50% was considered to demonstrate no obvious heterogeneity, and then a fixed-effect model (FEM) was applied to calculate the pooled OR, whereas a *P* value <0.05 or *I^2^*≥50% was considered to indicate the presence of heterogeneity, and then a random-effect model (REM) was adopted [20]. The associations of mtDNA haplogroups with OA were evaluated by calculating the pooled ORs and 95% CI. The significance of the pooled OR was assessed by Z test. Subgroup analysis of country was performed to explore the origin of heterogeneity when there is severe between-study heterogeneity. To identify the stability of the results, we conducted a sensitivity analysis by removing an individual study each time. Revman 5 was used for majority of the analysis performed here and STATA version 11.0 was applied to evaluate the publication bias. *P*<0.05 was considered statistically significant.

## Results

### Characteristics of the included studies

**Figure 1:**
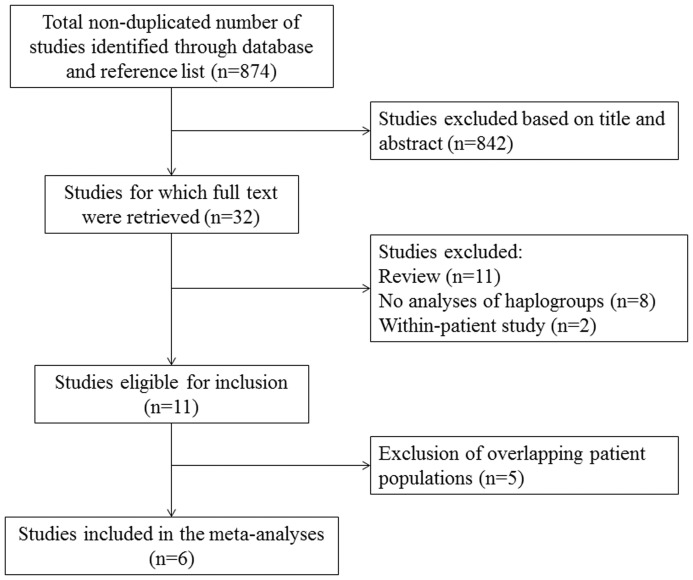
Flow chart of study selection for meta-analysis of mtDNA haplogroups and risk of OA.

The database and reference list search identified a total of 874 non-duplicated articles (Fig. 1). No non-published data that met our eligibility criteria were found. Through reading the titles and abstracts, 32 potentially relevant articles were remained for further full-text assessment. Due to the reasons listed in the flow chart, 11 remaining articles were initially included, of which, 5 articles were excluded because of overlapping patients populations [21]–[25]. Finally, 6 studies were included in our meta-analysis.

American College of Rheumatology (ACR) criteria [26] were adopted to make the diagnosis of OA in all the included studies, except the study by Hudson et al. [18], in which the radiographic evidence of disease (Kellgren-Lawrence grade ≥2) or clinical evidence of disease to a level requiring joint replacement was used. The included studies were all case-control studies and generated information for 10590 cases diagnosed with hip or knee OA and 7161 controls. Both the OA and control groups in each study contained female and male cases. According to the reported mtDNA haplogroups that probably correlated with OA risk, we investigated the relationship between haplogroups J, T, G and B and the risk of OA respectively in this meta-analysis. For haplogroups J and T, Caucasian population was recruited; while for haplogroups G and B, East Asian population was examined.

The detailed characteristics of the included studies are shown in the Table 1.

**Table 1:** Characteristics of studies included in meta-analysis.

Study	Public year	Country	Ethnicity	Mean age yr		Type of OA	Sample size		Method of genotype	Haplogroups affecting OA	Controlled confounders	NOS score
				OA	control		OA	control				
Rego [13]	2008	Spain	Caucasian	68	61	Knee	457	262	PCR-RFLP and SBE	J	None	5
Rego [14]	2010	Spain	Caucasian	69	69	Hip	550	505	PCR-RFLP and SBE	J	Gender, age, smoking	7
Fernandez [17]	2011	Spain	Caucasian	69	52	Hip	79	166	PCR-RFLP and SBE	J	Diagnosis, gender, age	7
Hudson [18]	2013	UK	Caucasian	66	NA	Hip, Knee	7393	5122	Illumine human610 platform	None	Gender, joint, case ascertainment method	7
Soto [16]	2014	UK	Caucasian	73	71	Hip, Knee	453	280	PCR-RFLP and SBE	T	Age, gender	7
Soto [16]	2014	Spain	Caucasian	69	66	Hip, Knee	1471	406	PCR-RFLP and SBE	J	Age, gender	7
Fang [15]	2014	China	Asian	62	55	Knee	187	420	PCR and sequencing	G, B	Age, gender	8

NA: not available.

### Quality assessment results

The details of the quality evaluation for every study are shown in Table 1. The average NOS score was 6.9. The most common biases were representativeness of the cases and selection of controls.

### Meta-analysis results

**Figure 2:**
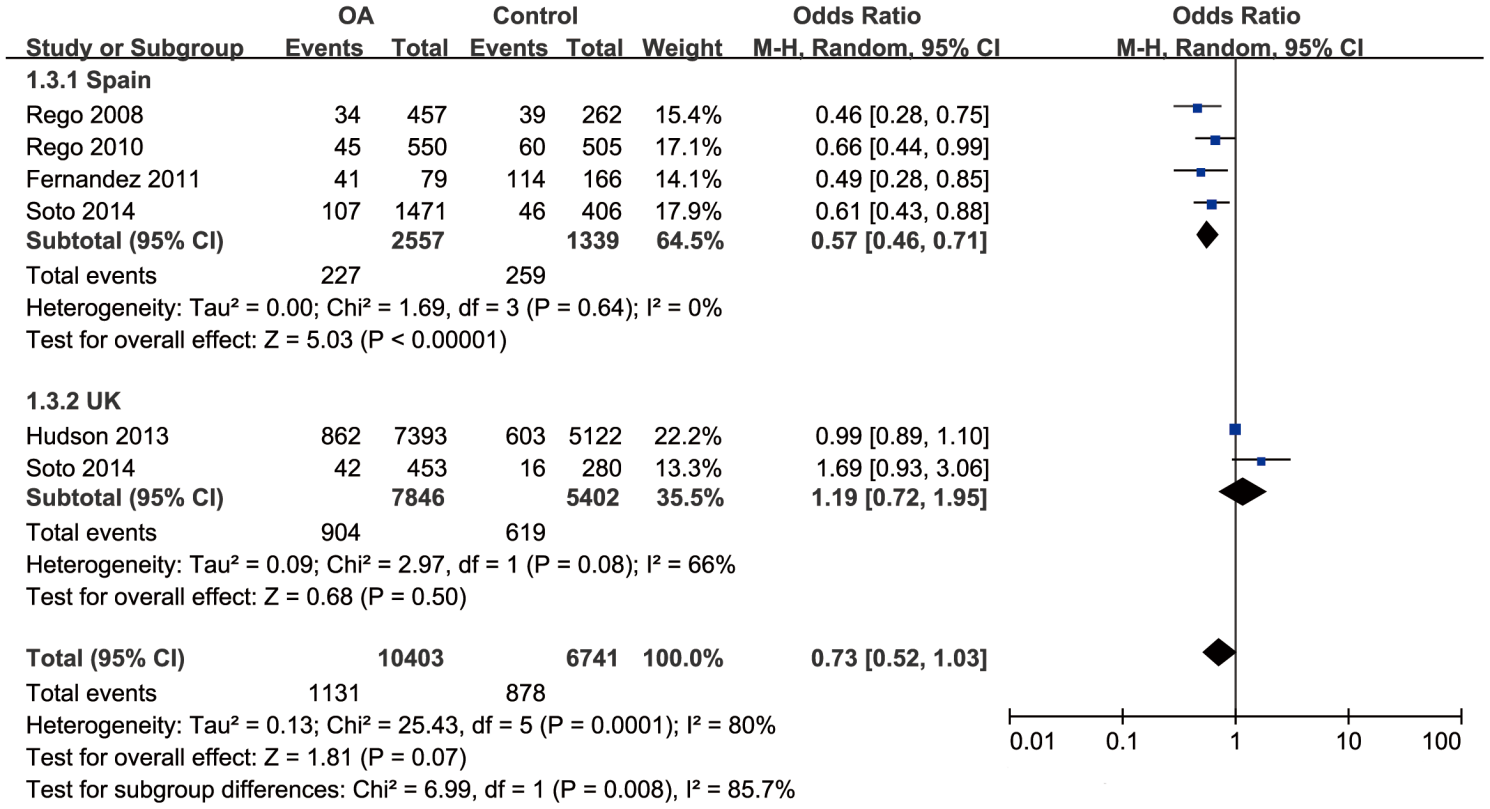
Forest plot of subgroup meta-analysis of the association between mtDNA haplogroup J and risk of OA.

**Figure 3:**
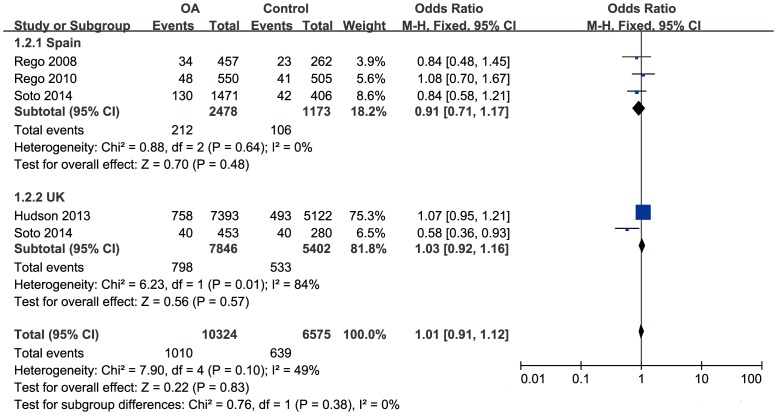
Forest plot of subgroup meta-analysis of the association between mtDNA haplogroup T and risk of OA.

Since severe between-study heterogeneity for haplogroup J (*I^2^* = 80%, *P*<0.01) was revealed, a random effect model was selected. Overall, the results showed that there was no significant association between haplogroup J and the risk of OA (OR = 0.73; 95%CI: 0.52–1.03, *P*>0.05) (Fig. 2). The subgroup analysis stratified by country revealed that there was a significant association in Spain population (OR = 0.57, 95%CI: 0.46–0.71, *P*<0.01), but no significant association in UK population (OR = 1.19, 95%CI: 0.72–1.95, *P*>0.05). No significant correlation was found between haplogroup T and risk of OA (OR = 1.01, 95%CI: 0.91–1.12, *P*>0.05), with moderate between-study heterogeneity (*I^2^* = 49%, *P*>0.05) (Fig. 3). Further subgroup analysis confirmed the irrelevance between them.

**Figure 4:**
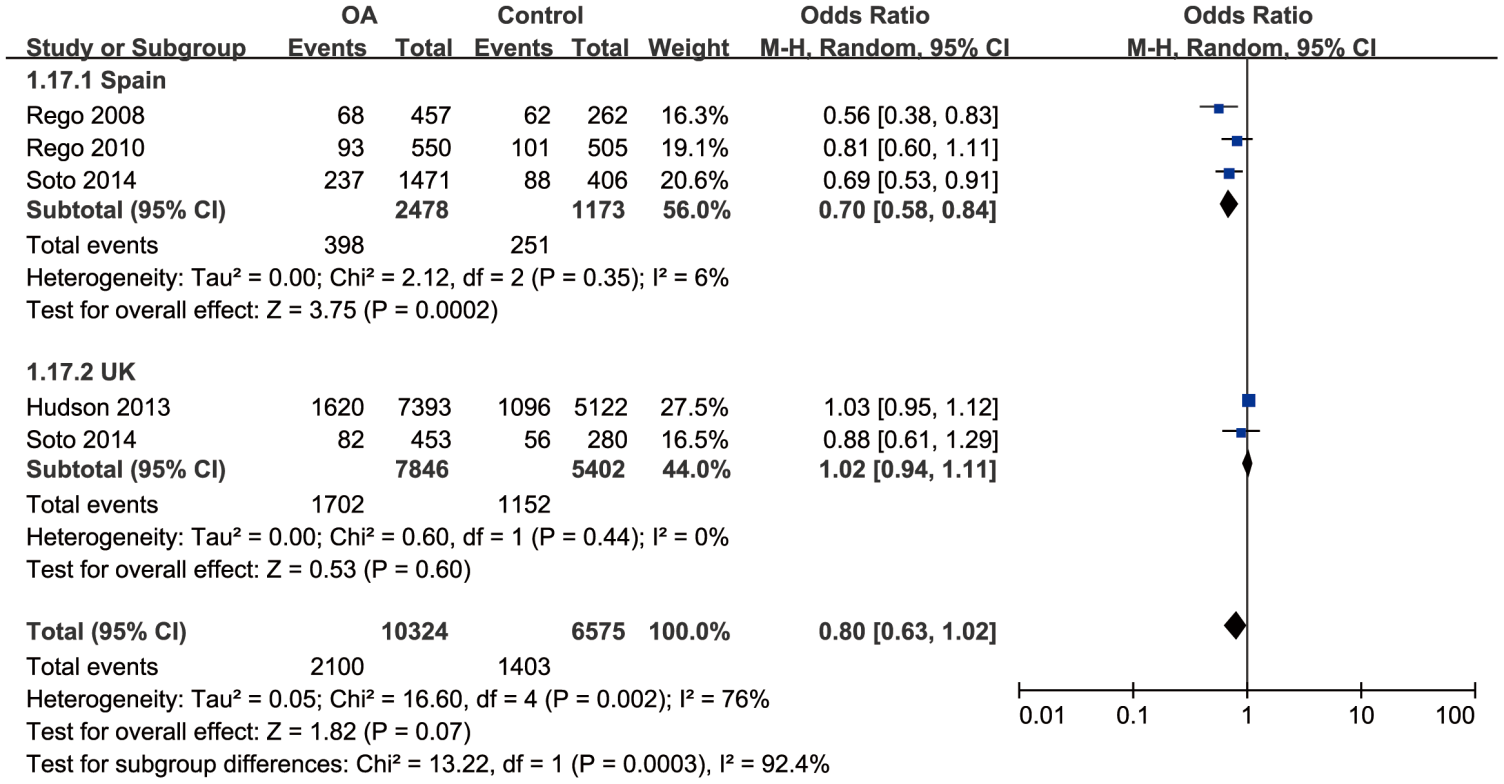
Forest plot of subgroup meta-analysis of the association between mtDNA cluster TJ and risk of OA.

**Figure 5:**
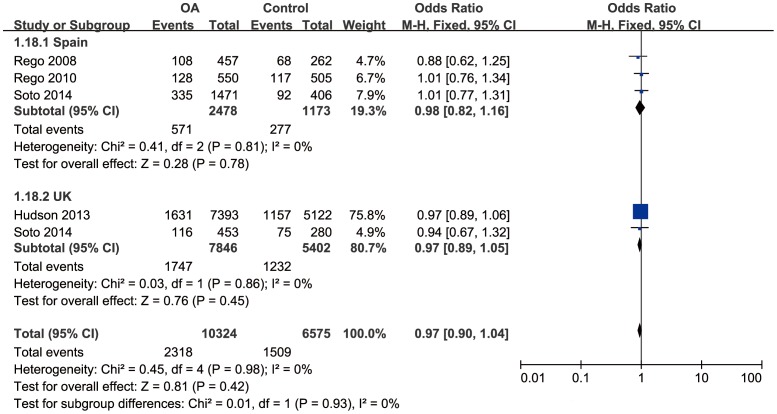
Forest plot of subgroup meta-analysis of the association between mtDNA cluster KU and risk of OA.

**Figure 6:**
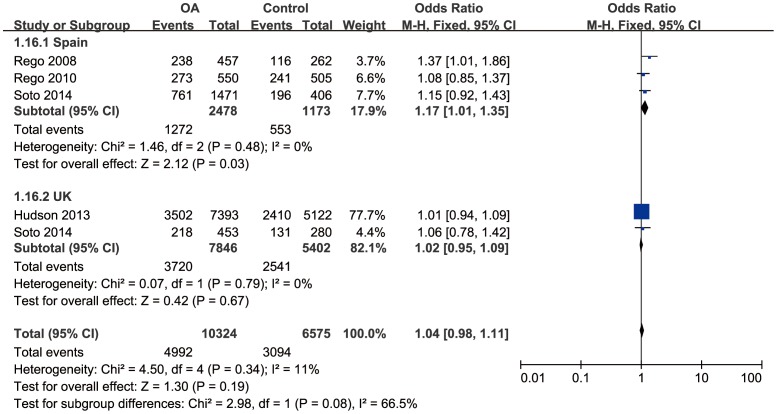
Forest plot of subgroup meta-analysis of the association between mtDNA cluster HV and risk of OA.

Considering that the mtDNA haplogroup effects may be due to the common features shared by the clusters, we compared the frequency of the clusters HV, KU and TJ between OA and control groups. Since there was severe between-study heterogeneity for cluster TJ (*I^2^* = 76%, *P*<0.05), subgroup analysis stratified by country was performed. There was a significant correlation between cluster TJ and OA in Spain population (OR = 0.70, 95%CI: 0.58–0.84, *P*<0.05), although no significant correlation in UK population (OR = 1.02, 95%CI: 0.94–1.11, *P*>0.05) (Fig. 4). No significant association was found between the cluster HV/KU and OA (OR = 1.04, 95%CI: 0.98–1.11, *P*>0.05/OR = 0.97, 95%CI: 0.90–1.04, *P*>0.05) with mild between-study heterogeneity (*I^2^* = 11%, *P*>0.05/*I^2^* = 0%, *P*>0.05). Further subgroup analysis confirmed the irrelevance between cluster KU and OA (Fig. 5). A marginal correlation between cluster HV and OA arose in Spain population (OR = 1.17, 95%CI: 1.01–1.35) (Fig. 6).

### Sensitivity analysis

Sensitivity analysis was performed by removing any study at one time. For haplogroup J, once a study from UK population [16], [18] was removed, the association between haplogroup J and risk of OA emerged, with ORs and 95%CIs 0.67 (0.46–0.98) [18] and 0.65 (0.45–0.92) [16] respectively; however, removing any study from Spanish population did not impact on the significance of results, with ORs and 95%CIs ranging from 0.75 (0.50–1.11) to 0.80 (0.57–1.12). For cluster TJ/HV, when the study by Hudson et al [18] was removed, there was a significant correlation with ORs and 95%CIs 0.73 (0.62–0.86)/1.15(1.01–1.30). For haplogroup T/cluster KU, the pooled effect was stable and was not influenced significantly by any study, with ORs and 95%CIs ranging from 0.83 (0.66–1.03) to 1.04 (0.94–1.16)/from 0.97(0.90–1.04) to 0.97(0.83–1.13).

### Publication bias

**Figure 7:**
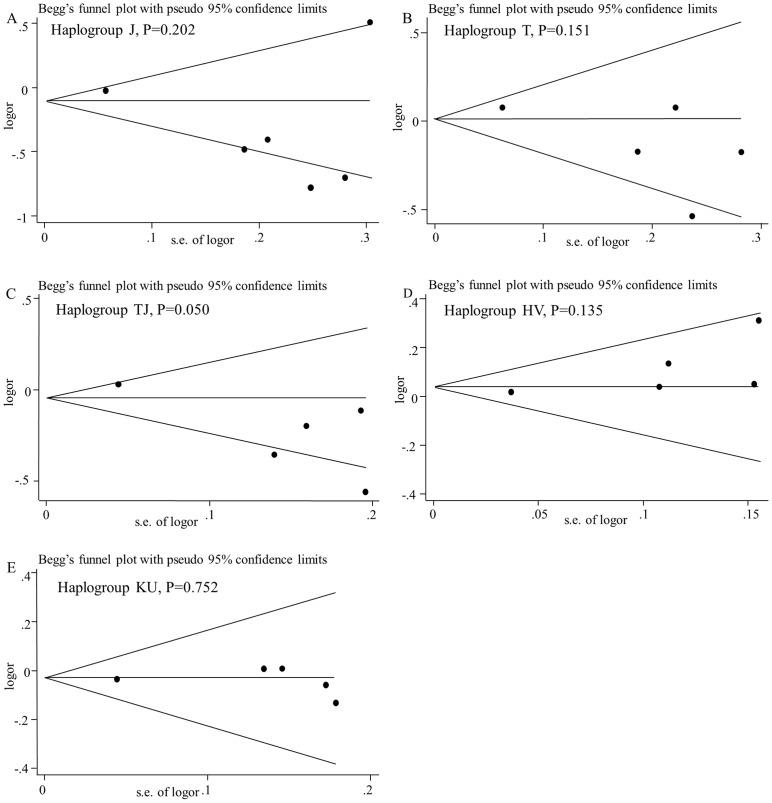
Begg's funnel plots with the Egger's test for publication bias of mtDNA haplogroups and risk of OA. Studies will be distributed symmetrically beside the horizontal line in absence of publication bias.

Egger's regression test and Begg's funnel plots were applied to evaluate the potential publication bias and suggested no severe publication bias (for haplogroup J: *P* = 0.202 and *P* = 0.707 separately; for haplogroup T: *P* = 0.151 and *P* = 0.462 separately; for cluster TJ: *P* = 0.050 and *P* = 0.462 separately; for cluster HV: *P* = 0.135 and *P* = 0.086 separately; for cluster KU: *P* = 0.752 and *P* = 0.221 separately) (Fig. 7).

For haplogroup TJ, the Egger's test showed that the publication bias was suspected with *P* = 0.05, so the trim and fill analysis was carried out to evaluate the impact of any asymmetry. However, the pooled estimate was not changed.

## Discussion

OA is a major cause of pain and impaired mobility in the elderly, and as the aging of population in modern society OA becomes an increasingly important public-health problem. OA is a multi-factor disease with both genetic and environmental causes [27]. It was reported that there was mtDNA deletion mutation and mtDNA damage [28]–[30] in the articular chondrocytes of OA, indicating an association between OA and mtDNA mutation [31]. In 2008 an association between mtDNA haplogroup J and OA was found, which proposed that haplogroup J protected against OA [13]. Later some studies demonstrated the association between mtDNA haplogroup J and risk of OA [14], [17]. However, a large population study in 2003 proposed no evidence of an association between all the haplogroups in Europe and OA. It is quite urgent to ascertain the correlation between haplogroups and OA, and the studies published recently made it possible to carry out a meta-analysis to elucidate this problem. To our best knowledge, this is the first meta-analysis addressing the association between mtDNA haplogroups and the risk of OA.

In this study we found that there was an association between haplogroup J/cluster TJ and risk of OA once a study from UK population was removed. Subgroup analysis also revealed that there was a significant association between haplogroup J/cluster TJ and risk of OA in Spain population, but not in UK population. It seems that there is a marginal correlation between cluster HV and risk of OA in Spain population. The investigation in Asian population indicated that haplogroup G was a risk factor and haplogroup B was protective [15]. All above suggests that mtDNA haplogroups do link to the risk of OA; however, the different ethnic and geological factors have a great influence on the association.

Considering the discrepancies of the association between mtDNA haplogroups and OA in different populations, several probabilities may be taken into account. Firstly, mtDNA haplogroup J consists of two major clades that can be further divided into more clades [32]. The distribution of mtDNA haplogroups were influenced by the past climate events [33], [34] and the constitution of the subhaplogroups of haplogroup J in different regions may be different. Seemingly some specific subhaplogroups function in the protection of OA [14], so the different constitution of subhaplogroups in different countries leads to the inconsistent results. Secondly, mutation of mtDNA may be complicated with epigenetic modification of nuclear genome, which contributes to various phenotypes in the same mtDNA mutation [35], [36]. The crosstalk among mtDNA, nucleus genome, epigenetics, and environment may lead to the different contribution of specific mtDNA haplogroups in OA in different populations.

The exact mechanism underlying the association of mtDNA haplogroups and OA risk remains unclear. Mitochondria produce ROS that play an important role in apoptosis [37]. Chondrocytes in OA exhibit a production increase of ROS and chronic oxidant stress, which may increase chondrocyte death and finally lead to OA [38]. Haplogroup J carriers harbor mtDNA missense mutations that partially uncouple OXPHOS, leading to less ATP production and meanwhile decreasing mitochondrial ROS generation by increasing the oxidation of electron transport chain (ETC). This reduction might decrease the oxidative damage to the cell, protect against apoptosis [39], [40], reduce cartilage degradation, and avoid the risk of OA.

The present study has some limitations. First, all the included studies were case-control studies, which tended to contain selection bias. In addition, the included studies adopted various control groups, including hospital controls with ailments unrelated to OA [14], [15], [17], population with joint fracture [16], university population [17], birth cohort [18], and blood service control groups [18]. Therefore, it is difficult to control the confounding factors and selection bias. Second, the publication bias was suspected for haplogroup TJ. Publication bias may result in an inaccurate estimation of the association between haplogroup TJ and OA. However, the results remained unchanged after “trim and fill” analysis, indicating that the publication bias of haplogroup TJ was not severe. Third, only two studies investigated the mtDNA subhaplogroups [14], [15]. Since we had insufficient data of the contribution of various subhaplogroups, we could not perform the meta-analysis on subhaplogroups data. Fourth, the major geographic cohorts involved in investigating the correlation between OA and mtDNA haplogroups are Spain, England, and China, which are still quite limited. For haplogroups G and B, only one study could be included [15]. More investigations are required to clarify the authentic association and the future studies should pay more attention to geographical difference.

In conclusion, our meta-analysis suggests that mtDNA haplogroup J and cluster TJ correlate with the risk of OA in Spanish population; however, the association in other populations is still not clear. Further investigations in different regions, with satisfying controls, and containing the data of mtDNA subhaplogroups are required to clarify the correlation and the underlying mechanism.

## Supporting Information

Checklist S1 **The PRISMA checklist.**
(DOC)

Methods S1 **Search strategies in PubMed, Web of Science, SDOS and CNKI.**
(DOCX)
