# Supplementary material for: Role of mtDNA Haplogroups in the Prevalence of Osteoarthritis in Different Geographic Populations: A Meta-Analysis
Source: PLoS One. 2014 Oct 23;9(10):e108896. doi: 10.1371/journal.pone.0108896 (PMC4207685; doi:10.1371/journal.pone.0108896)
Supplement: Methods S1 — Search strategies in PubMed, Web of Science, SDOS and CNKI. (DOCX) [file pone.0108896.s002.docx]

**Pubmed**

#1 *osteoarthritis*

#2 *mtDNA* OR “*mitochondrial DNA*”

#3 *haplogroup* OR *haplotype* OR *genotype* OR “*genetic predisposition*” OR *SNP* OR *polymorphism** OR *variant** OR “*genetic susceptibility*” OR *genetics OR allele*

#4 #1 AND #2 AND #3

**Web of Science**

# 1 TOPIC: (*Osteoarthritis*) Indexes=SCI-EXPANDED, SSCI, A&HCI, CPCI-S, CPCI-SSH, CCR-EXPANDED, IC Timespan=All years

# 2 TOPIC: (*mtDNA*) OR TOPIC: (“*mitochondrial DNA*”) Indexes=SCI-EXPANDED, SSCI, A&HCI, CPCI-S, CPCI-SSH, CCR-EXPANDED, IC Timespan=All years

# 3 TOPIC: (*haplogroup*) OR TOPIC: (*haplotype*) OR TOPIC: (*genotype*) OR TOPIC: (“*genetic predisposition*”) OR TOPIC: (“*genetic predisposition*”) OR TOPIC: (*SNP)* OR TOPIC: (*polymorphism**) OR TOPIC: (*variant**) OR TOPIC: ("*genetic susceptibility*”) OR TOPIC: (*genetics*) OR TOPIC: (*allele*) Indexes=SCI-EXPANDED, SSCI, A&HCI, CPCI-S, CPCI-SSH, CCR-EXPANDED, IC Timespan=All years

#4 #1 AND #2 AND #3 Indexes=SCI-EXPANDED, SSCI, A&HCI, CPCI-S, CPCI-SSH, CCR-EXPANDED, IC Timespan=All years

**SDOS**

#1 *Osteoarthritis* [All Sources(- All Sciences -)]

#2 *mtDNA* OR “*mitochondrial DNA*” [All Sources(- All Sciences -)]

#3 (*haplogroup* OR *haplotype* OR *genotype* OR “*genetic predisposition*” OR *SNP* OR *polymorphism** OR *variant** OR “*genetic susceptibility*” OR *genetics* OR *allele*) [All Sources(- All Sciences -)]

#4 (*Osteoarthritis*) AND (*mtDNA* OR “*mitochondrial DNA*”) AND ((*haplogroup* OR *haplotype* OR *genotype* OR “*genetic predisposition*” OR *SNP* OR *polymorphism** OR *variant** OR “*genetic susceptibility*” OR *genetics* OR *allele*)) [All Sources(- All Sciences -)]

**CNKI**

#1 *骨性关节炎* (*Osteoarthritis*)

#2 *线粒体DNA* (*mitochondrial DNA*)

#3 #1 AND #2
